# Supplementary material for: Content-rich biological network constructed by mining PubMed abstracts
Source: BMC Bioinformatics. 2004 Oct 8;5:147. doi: 10.1186/1471-2105-5-147 (PMC528731; doi:10.1186/1471-2105-5-147)
Supplement: Additional File 2 — The original results of the above study (non-essential files are deleted to keep the file size under the limit set by BMC bioinformatics). [file 1471-2105-5-147-S2.bz2 › chilibotAdditionalFile2/dip10/528ID9671457E10845/html/ESR1_HMGB1.html]

 


 **ESR1** and **HMGB1** 
  
Found 23 abstracts in PubMed, retrieved 10.  
 

 What does Google say? 
 PDF only 
| .edu only 

---

**Interactive relationship** (e.g. stimulation, inhibition, etc)

**Stimulatory relationship**- The high mobility group protein 1  [ **HMGB1** ]  enhances binding of the estrogen receptor  [ **ESR1** ]  DNA binding domain to the estrogen response element.  Ref: 9605929 Mol Endocrinol, 1998
- HMG 1  [ **HMGB1** ]  stimulates estrogen response element binding by estrogen receptor  [ **ESR1** ]  from stably transfected HeLa cells.  Ref: 10194768 Mol Endocrinol, 1999
**Neutral relationship**- Furthermore, the finding of two estrogen responsive elements within intron 1 is relevant as they indicate a direct mechanism of HMG1  [ **HMGB1** ]  up regulation by estrogen making the presence of an estrogen receptor  [ **ESR1** ]  a significant marker for a combined treatment of special tumor types with estrogen and the anticancer drugs cisplatin or carboplatin.  Ref: 11299752 Anticancer Res< MedlineTA>Anticancer Res, 1999

**Non-interactive relationship** (e.g. studied together, co-existance, homology, etc.)

- We have examined the ability of the high mobility group protein 1  [ **HMGB1** ]  HMG1  [ **HMGB1** ]  to alter binding of the estrogen receptor  [ **ESR1** ]  DNA binding domain DBD to the estrogen response element ERE.  Ref: 9605929 Mol Endocrinol, 1998
- However, expression of a reporter driven by the rat HMG  [ **HMGB1** ]  CoA reductase promoter was induced by estrogen treatment after transient transfection into the breast cancer cell line MCF 7 cells but not in hepatic cell lines expressing estrogen receptor  [ **ESR1** ] .  Ref: 10446899 Mol Endocrinol, 1999
- Regulation of the expression of HMG1  [ **HMGB1** ] , a co activator of the estrogen receptor  [ **ESR1** ] .  Ref: 11299752 Anticancer Res< MedlineTA>Anticancer Res, 1999
- Because HMG1  [ **HMGB1** ]  dramatically enhanced estrogen receptor  [ **ESR1** ]  DBD binding to the ERE, and the DBD is the most highly conserved region among the nuclear receptor superfamily members, HMG1  [ **HMGB1** ]  may function to enhance binding of other nuclear receptors to their respective response elements and act in concert with coactivator proteins to regulate expression of hormone responsive genes.  Ref: 9605929 Mol Endocrinol, 1998
- 1 009 1019 that HMG1  [ **HMGB1** ]  promotes the binding of the estrogen receptor  [ **ESR1** ]  to the estrogen response element and further reinforce our believe that HMG1  [ **HMGB1** ]  plays a significant role in estrogen induced gene expression.  Ref: 9633537 Exp Cell Res, 1998
- This variation may contribute to the different response, of estrogen receptor  [ **ESR1** ]  positive breast tumours to endocrine therapy, making  **HMGB1**  a marker of considerable clinical interest.  Ref: 11911263 Anticancer Res< MedlineTA>Anticancer Res, 1998
